# Supplementary material for: Expression, Prognostic Value and Correlation with HPV Status of Hypoxia-Induced Markers in Sinonasal Squamous Cell Carcinoma
Source: J Pers Med. 2023 Apr 29;13(5):767. doi: 10.3390/jpm13050767 (PMC10222519; doi:10.3390/jpm13050767)

Supplementary figure S1

Kaplan-Meier survival curve showing overall survival based on GLUT-1 expression.

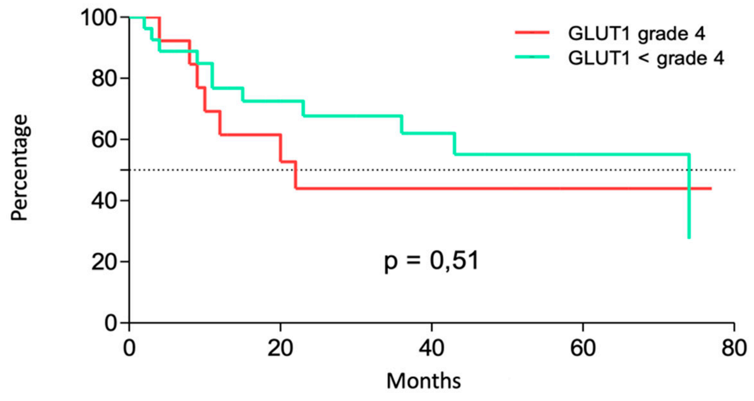

Supplementary figure S2

Kaplan-Meier survival curve showing overall survival based on VEGF expression.

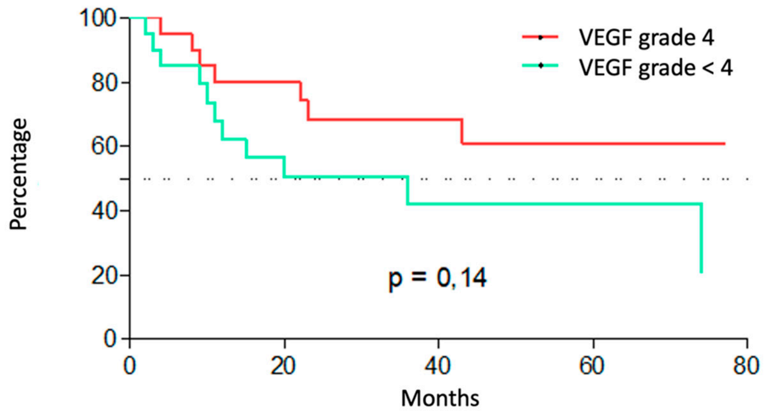

Supplementary figure S3

Kaplan-Meier survival curve showing overall survival based on HIF-1α expression.

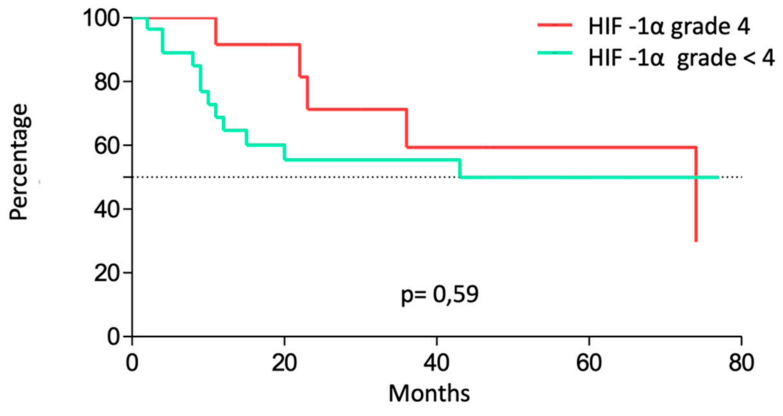

Supplementary figure S4

Kaplan-Meier survival curve showing overall survival based on HPV positivity.

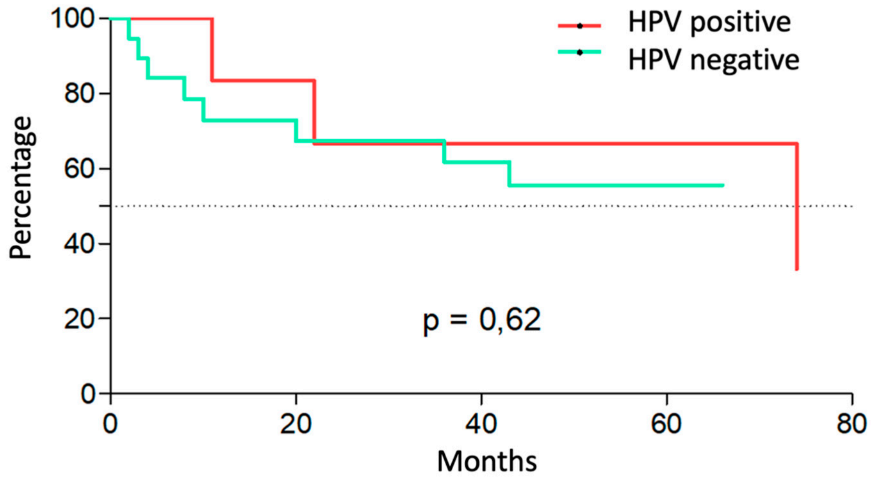

Supplement: Supplementary file 1 [file jpm-13-00767-s001.zip › jpm-2344004-supplementary.pdf]
